# Immunoglobulin A Nephropathy With Extracapillary Proliferation and Positive Anti-Neutrophil Cytoplasmic Antibodies: A Rare Cause of Dual Glomerulonephritis

**DOI:** 10.7759/cureus.11068

**Published:** 2020-10-20

**Authors:** Jose C Alvarez, Joaquin Rodelo, Luis F Arias, Cristian Álvarez

**Affiliations:** 1 Internal Medicine, University of Antioquia, Medellin, COL; 2 Nephrology, Nephrology Unit, San Vicente Foundation University Hospital, Medellin, COL; 3 Pathology, University of Antioquia, Medellin, COL; 4 Internal Medicine, University of Sucre, Sincelejo, COL

**Keywords:** anti-neutrophil cytoplasmic antibody-associated vasculitis, antineutrophil cytoplasmic autoantibodies, iga glomerulonephritis, hiv diseases

## Abstract

The coexistence of immunoglobulin A (IgA) nephropathy and associated pauci-immune anti-neutrophil cytoplasmic antibody (ANCA) glomerulonephritis represents a rare concurrence of two common forms of glomerulonephritis; the pathogenesis, treatment, and prognosis of this dual glomerulopathy are not well described. This illustrative case can present this association in an HIV-positive patient and how despite the coexistence of these two entities, the patient had control of his kidney disease with low doses of steroids, contrary to the different reports of cases in the literature in which the treatment is often more aggressive. In this case report, we review the literature on this dual glomerulonephritis and confront clinical and treatment aspects regarding the different clinical cases reported in the databases.

## Introduction

Although immunoglobulin (Ig) A associated nephropathy and anti-neutrophil cytoplasmic antibody (ANCA) associated pauci-immune glomerulonephritis are not uncommon, their coexistence is quite rare. A prevalence of such coexistence has been previously reported to be 0.2-2% [1-4]. IgA nephropathy is the most common primary glomerulopathy [5], with a prevalence on renal biopsy of 3-16% [6,7]; it is characterized by IgA deposits, which can be associated with subtle IgG or IgM staining in glomerular mesangium [5]. ANCA-associated glomerulonephritis is usually depicted by pauci-immune extracapillary proliferation [8].

Here we present the case report of an HIV-positive patient with adequate viral control who presented with hematuria, pyuria, and subnephrotic proteinuria without any affection in the glomerular filtration rate (GFR); the renal biopsy showed IgA deposits, extracapillary proliferation, and positive anti-myeloperoxidase antibodies. The clinical implication of the coexistence of both clinical entities is discussed.

## Case presentation

**Figure 1:**
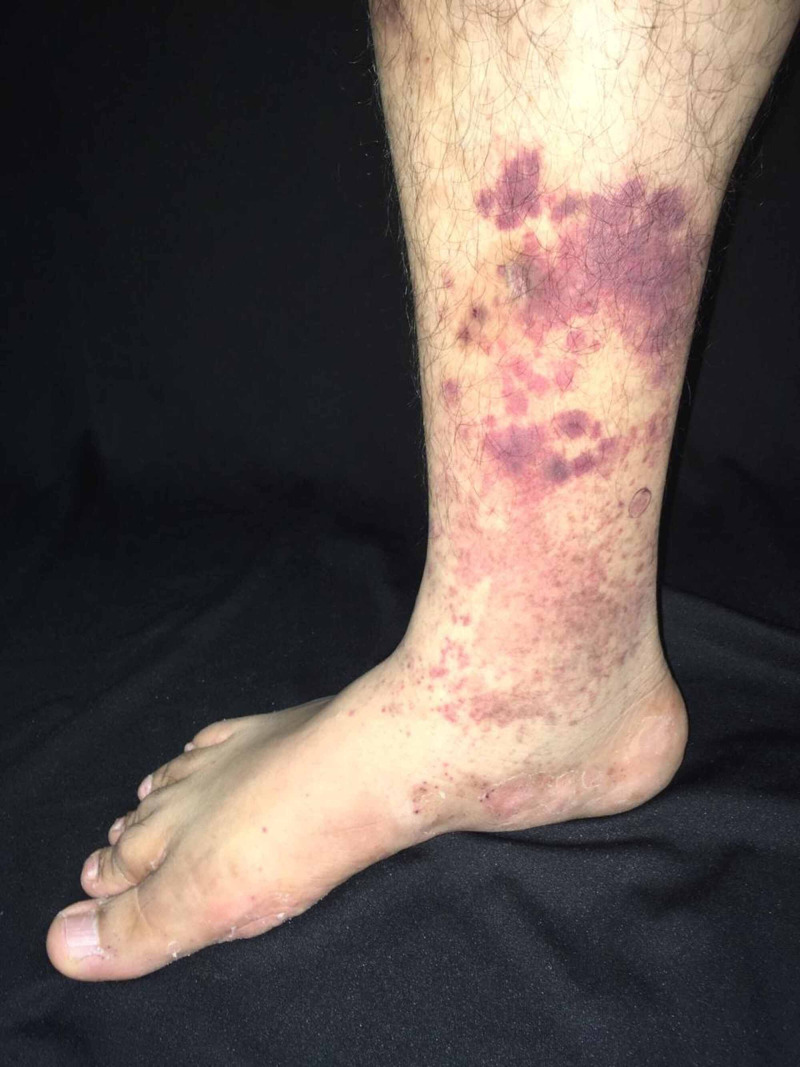
Purpuric lesions in the right lower limb.

A 40-year-old male with a personal history of HIV infection, CD4 cells of 348, and undetectable viral load treated with tenofovir/lamivudine and atazanavir/ritonavir presented after four months of bilateral knee pain with gait difficulties, erythematous-violaceous rash in the lower limbs, fever, and chills. He also had a history of adequately treated pulmonary tuberculosis, partially treated syphilis, and recurrent herpes in the lumbar region. On physical examination, purpuric lesions were observed in the lower limbs (Figure 1). Laboratory data were as follows: white blood count of 9,600 cells/mcL, neutrophil count of 7,600 cells/μL, lymphocyte count of 1,700 cells/mm^3^, platelets of 249,000/μL, hemoglobin of 15.2 gm/dL, hematocrit of 43%, mean corpuscular volume of 95 fL, creatine kinase of 71 U/L, lactate dehydrogenase of 184 U/L, blood urea nitrogen of 14 mg/dL, and creatinine (Cr) of 0.85 mg/dL. Coagulation tests, infectious disease tests, and electrolytes were normal. Complement C3 was 155 mg/dL and C4 24 mg/dL. Anti-proteinase 3 (PR3) antibodies were negative, and anti-myeloperoxidase (MPO) antibodies were positive at a 42 titer. Anti-nuclear antibodies and extractable nuclear antigen antibodies were negative. Uroanalysis reported 500 mg of protein, blood of 250 (<3 per high power field), and white blood cells of 25 (<5 per high power field). Urine sediment showed 17 red blood cells per high power field (HPF), 10 white blood cells/HPF, and 18 granular and 1 hyaline casts/HPF; no bacteria were observed. Urine culture was negative. The 24-hour urine protein level was 2.9 grams.

**Figure 2:**
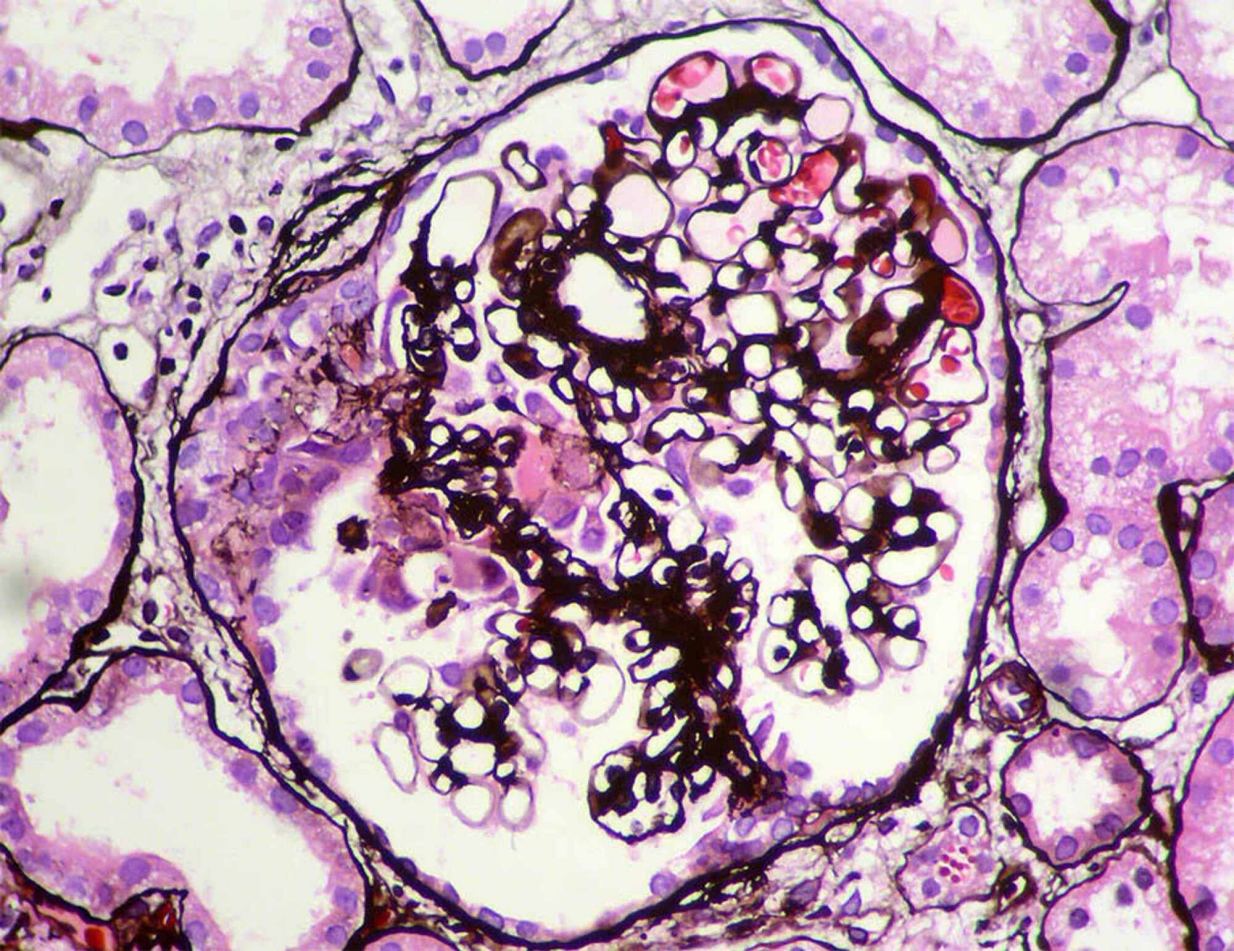
A glomerulus with a necrotizing segment, with rupture of the capillary walls and an adjacent crescent (left) (methenamine silver stain (X400).

Renal biopsy was performed, which showed that of the 32 glomeruli sampled for light microscopy, 6 contained cellular crescents, ranging from circumferential to segmental. In the underlying glomerular tuft, there was variable mesangial and segmental endocapillary hypercellularity with infiltrating leukocytes. One glomerulus had segmental fibrinoid necrosis. Five glomeruli were globally sclerotic. There was not tubular atrophy. Renal interstitium showed sparse mononuclear inflammatory infiltrate, and there were a few red blood cell casts associated with acute tubular injury. Vessels did not display arteritis (Figure 2).

**Figure 3:**
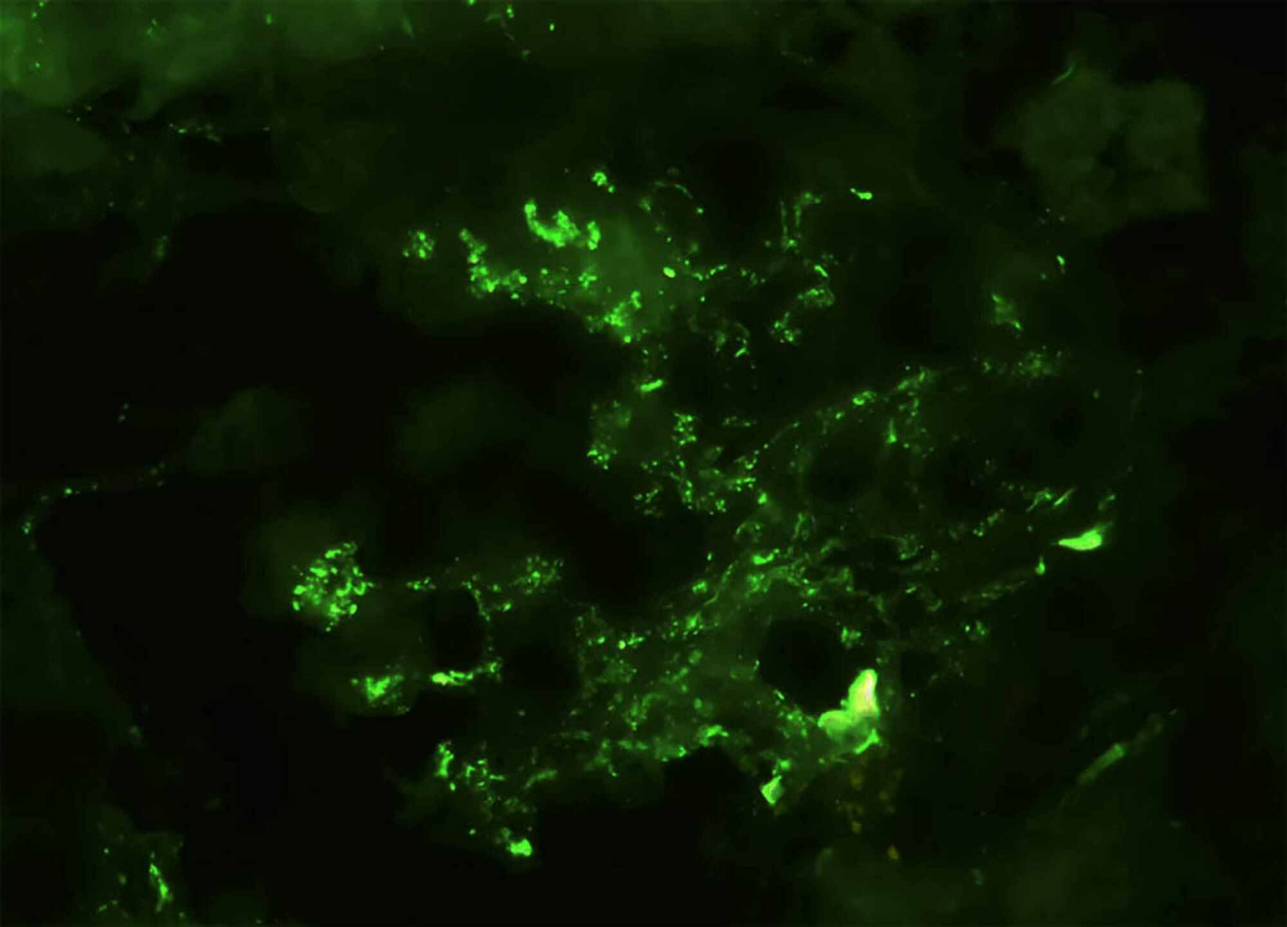
Kidney biopsy immunofluorescence, with direct immunofluorescence for C3 demonstrating mesangial positivity with some parietal segmental granules (X400).

**Figure 4:**
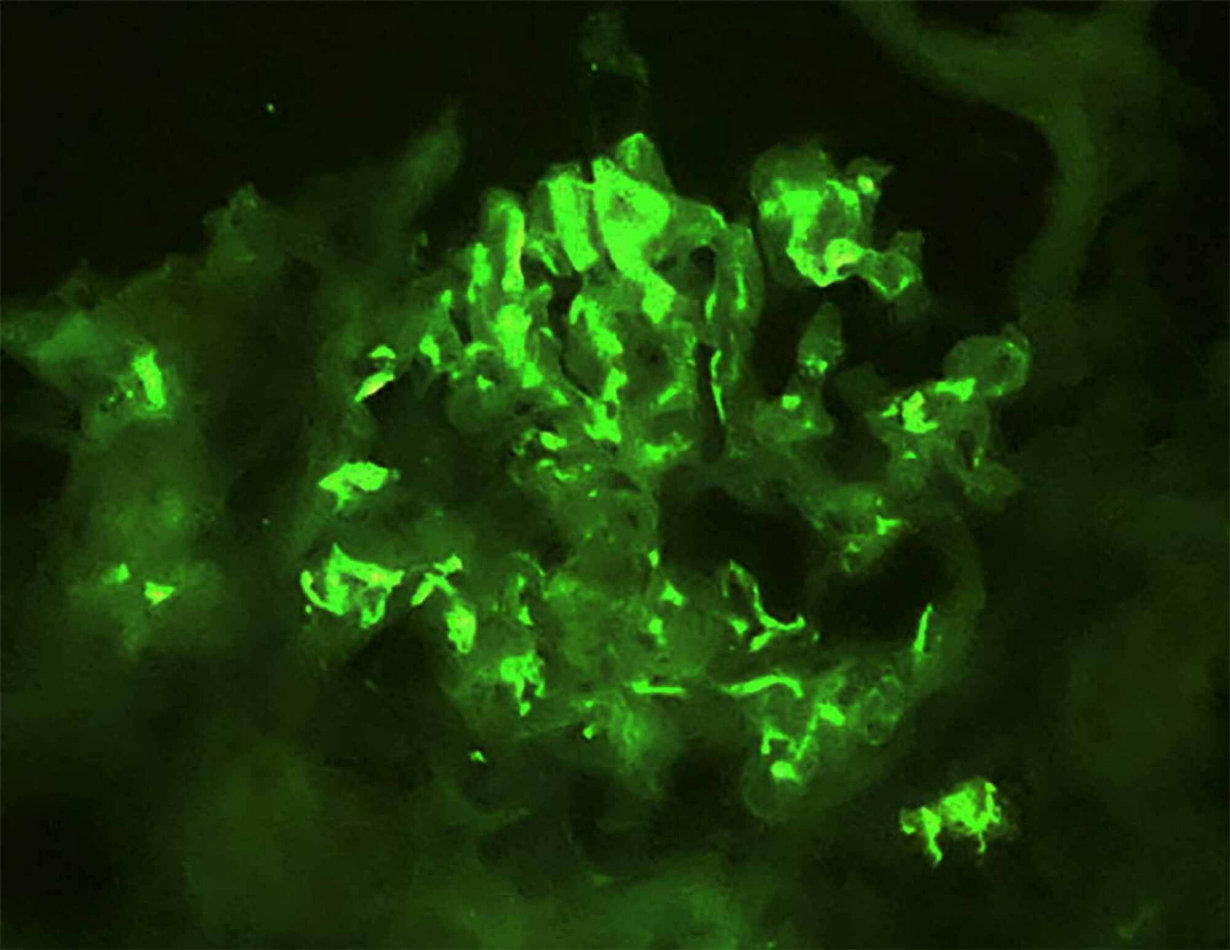
Kidney biopsy Immunofluorescence, with direct immunofluorescence for IgA demonstrating greater mesangial intensity and some subendothelial deposits (X400).

Among the six glomeruli sampled for immunofluorescence, four showed dominant 2-3+ positivity for IgA in a diffuse mesangial distribution, accompanied by 2+ C3. There was no glomerular staining for IgG, IgM, or C1q (Figures 3, 4).

The renal biopsy diagnosis was crescentic IgA nephropathy associated with anti-MPO antibody seropositivity. Additionally, skin biopsy of lower limbs lesions was performed, where a leukocytoclastic vasculitis with negative immunofluorescence was observed.

The patient was only treated with prednisolone 10 mg day and enalapril 20 mg day and discharged. Follow-up one month later showed preserved renal function (Cr of 0.78 and GFR of 117 by the MDRD equation), uroanalysis without active sediment, and 24-hour urine protein level of 300 mg. The patient was completely asymptomatic and without lesions skin.

## Discussion

In this patient with a personal history of well-controlled HIV, our attention was brought to the association between ANCA-associated glomerulonephritis and IgA nephropathy, which was linked to a skin biopsy with leukocytoclastic vasculitis findings, but no complement or antibodies deposits were observed.

The causal relationship between IgA nephropathy and ANCA-associated glomerulonephritis is unclear; nonetheless, most patients are diagnosed with a condition superposed to these two entities and usually have other extrarenal manifestations of vasculitis [4-8]. Coexistence of both diagnoses in renal biopsy can be considered a dual glomerulopathy. There are less than 20 cases reported, with four case series describing the association between IgA nephropathy and ANCA positivity [8].

Haas et al. reported a series of six patients with crescentic glomerulonephritis associated with mesangial IgA deposits and positive titers of ANCA antibodies [2]. In their study, four patients had anti-PR3 antibodies, one patient had anti-MPO antibodies, and one patient had both. Glomeruli presented with subtle mesangial and endocapillary hypercellularity compared to ANCA-negative IgA nephropathy patients who usually present with higher mesangial and endocapillary proliferation. Five patients had follow-up data available: three of them recovered renal function after cyclophosphamide and glucocorticoid treatment. The other two patients required dialysis therapy from the time of the diagnosis and afterwards. Our patient had no compromise of GFR, and no aggressive immunosuppressant or renal replacement therapy was required.

Bantis et al. described a series on eight patients, of which five had positive anti-MPO antibodies and three had positive anti-PR3 antibodies. Compared to ANCA-negative patients, those who presented with IgA extracapillary proliferation associated with positive ANCA had rapidly progressive glomerulonephritis and higher grade of renal insufficiency. Despite a similar intensity of IgA deposits, mesangial cellularity was higher in ANCA-negative patients compared to ANCA-positive patients. All of the eight patients with IgA extracapillary proliferation and ANCA-positive antibodies received aggressive therapy with cyclophosphamide and glucocorticoids; all of them had an improvement of renal function. In contrast, only a small minority of ANCA-negative patients received aggressive therapy (5/26 patients); the rest received glucocorticoids only, mycophenolate mofetil, or angiotensin-converting enzyme inhibitors. All of these patients had progressive renal failure after six months of follow-up. This study’s conclusion is that a less aggressive treatment could be the variable that led to such outcome.

It is uncertain if the coexistence of ANCA and IgA is a mere coincidence or if it is truly pathogenic. It’s possible that patients had preexistent IgA deposits in mesangium and then developed necrotizing lesions and crescents due to ANCA. This is inferred from an autopsy study that showed 4.8% mesangium IgA deposits without any history of renal disease [7]. In some patients with IgA nephropathy, the presence of ANCA antibodies may lead to a more aggressive glomerulonephritis with a higher percentage of necrosis and crescents; but, in other patients, the ANCA positivity may not be pathogenic. Based on a limited number of case reports, it’s unclear whether the type of ANCA (PR3 or MPO) may offer any clinical distinction.

Finally, such finding may be associated with HIV; nonetheless, few cases have been reported. The pathogenesis of Henoch-Schönlein purpura in HIV-infected patients is a hypothetical one: a higher number of circulating immune complexes associated with HIV infection has been suspected as the mechanism, but it has not been proven. HIV infection causes a polyclonal hypergammaglobulinemia with a particular elevation of IgG and IgA [9].

In our patient, a non-aggressive approach was chosen, with low doses of glucocorticoids and no additional immunosuppressant, given the history of a partially treated syphilis. He was discharged with no significant GFR impairment in the follow-up one month later.

## Conclusions

Concomitant IgA nephropathy with an ANCA-associated extracapillary component is an infrequent finding in clinical practice, with different outcomes in the case series previously reported. The pathophysiological mechanisms relating HIV with such entities or the renal outcome are unknown. Although rare, more patients have been reported with dual glomerulopathies with uncertain renal prognosis, such as our patient. In this case, the clinical expression of leukocytoclastic vasculitis without IgA deposit in immunofluorescence, but with an active urinary sediment and no GFR impairment and with the kidney biopsy previously described, required low doses of glucocorticoids and angiotensin-converting enzyme inhibitors.
